# Professor G. R. Treviranus

**Published:** 1838-01

**Authors:** 


### G. R. TREVIRANUS, OF BREMEN.

Gottfried Reinhard Treviranus was born in Bremen, February 1, 1776, of a respectable mercantile family. He was the eldest of eight children, three of whom only survive him, and one of whom (Ludolf Christian) is the distinguished professor of botany at Bonn, and was the able coadjutor of his brother in many of his works. G. R. Treviranus received his scholastic education in Bremen, where he shewed early talents and a decided inclination for science, although his family wished him to follow the mercantile profession. Having, however, selected medicine, he repaired to Göttingen, and pursued his studies from 1792 to 1796, with that indefatigable zeal which marked his whole life. He early shewed a taste for physiology, and indeed, in 1795, published anonymously a physiological essay, entitled "Ueber Nervenkraft und ihre Wirkungsart." In September, 1796, he took his degree of doctor; his thesis being on his favorite subject, viz. "De emendanda Physiologia." Shortly after his return to Bremen, he married; and, although he gave himself to the practice of medicine, he did not at first like it, on account of the interruption it created to his studies. Still he followed it, as well on account of its emoluments as from its intimate connexion with his cherished pursuits. He accordingly divided his time as follows: In the early part of the forenoon he visited his patients, and gave the remainder of it to dissections and microscopical observations; the spare hours of the afternoon he devoted to reading, those of the evening to composition,—seldom or ever leaving his native town. In this quiet and philosophical manner he passed forty years, happy in his pursuits; in the prosecution of which he may be said to have died, as only ten days before his death he visited some patients, and busied himself in correcting the proofs of one of his physiological writings.

Soon after his return from the university, viz. in 1797, he was appointed professor of medicine and mathematics in the Lyceum of Bremen. The same year he published his first work, the first part of his "Physiologische Fragmente;" and which was followed by numerous well-known and most valuable publications, either in the journals or separately, during the remainder of his busy life. The following are his principal works:

1. Physiologische Fragmente. Two vols. 8vo.—Hannov. 1797-99.
2. Biologie, oder Physiologie der lebenden Natur. Six vols. 8vo.—Götting. 1802-1822.
3. Ueber d. innern Bau der Arachniden.—Nurnb. 4to. 1812.
4. Beiträge zur Anatomie und Physiologie der Sinneswerkzeuge des Menschen und Thiere. Fol. 1828.
5. Beiträge zur Aufklärung der Erscheinungen und Gesetze des organischen Lebens. Two vols. 8vo. 1831-33.
6. Vermischte Schriften, anatom. und physiol. inhalts, (with L. C. Treviranus.) Four vols. 8vo. 1816-1820.

From the year 1824 until his death, Treviranus edited, in conjunction with his distinguished friend Tiedemann, the celebrated "Zeitschrift für Physiologie," of which five volumes have been published, and in each of which one or more of our author's excellent papers appeared.

Treviranus long laboured under a morbid state of the lungs, which had for several years before his death often put on a serious character, especially during winter. It was an attack of the same affection, a low nervous pneumonia, which terminated his life on the 16th February, 1837.

Of the great merits of Treviranus as a physiologist, it is hardly necessary to speak: they are universally known and acknowledged. He possessed at once a philosophical and a classical mind; and he was as distinguished for rigid honesty as for unwearied perseverance. He was much and justly valued as a physician. He was no less estimable in all the civil and social relations: he was an excellent husband and father, a true and faithful citizen.*

